# Physicochemical and aromatic characterization of carob macerates produced by different maceration conditions

**DOI:** 10.1002/fsn3.1374

**Published:** 2020-01-09

**Authors:** Karla Hanousek Čiča, Jasna Mrvčić, Siniša Srečec, Katarina Filipan, Marijana Blažić, Damir Stanzer

**Affiliations:** ^1^ Faculty of Food Technology and Biotechnology Zagreb Croatia; ^2^ Križevci College of Agriculture Križevci Croatia; ^3^ Karlovac University of Applied Sciences Karlovac Croatia

**Keywords:** aroma, carob, ethyl hexanoate, liqueur, macerate, phenolic compounds

## Abstract

Carob liqueur is an alcoholic drink (minimum 15% v/v of ethanol and 100 g/L of sugar) typical for the Mediterranean countries. In the current work, carob macerate produced by maceration of carob pods in hydroalcoholic base at different maceration conditions was characterized for the first time based on its aroma compounds/profile, physicochemical parameters, and chromatic characteristics. The results confirm the migration process of bioactive compounds, aroma compounds, and sugars flowing from the carob pod to the hydroalcoholic base. Changes in ethanol concentration modify the physical properties of the solvent and influence the phenolic and aroma compounds extraction, color, and acidity of the obtained samples. The higher content of phenolic compounds was determinate in the samples obtained in the darkness. The amounts of phenols were in the range of some red fruit liqueurs or walnut liqueurs, and sugars (mostly sucrose) ranging between 96 and 107 g/L. Twenty‐six (out of total 94) aroma compounds were detected in all samples, of which 17 esters, 3 alcohols, 4 ketones, and 2 acids. Low molecular weight ethyl esters, ethyl hexanoate, ethyl 2‐methyl propanoate, ethyl octanoate, ethyl benzoate, ethyl butanoate, and ethyl cinnamate, were the most abundant. Carob pod maceration in 50% v/v hydroalcoholic base (1:5 solid to liquid ratio) in darkness at room temperature during 8 weeks can be recommended as optimal maceration conditions for production of the aromatic carob macerate with functional properties.

## Graphical abstract

The results confirm migration process such as: bioactive compounds, aroma compounds, and sugars flows from the carob pod to the alcoholic base. Changes in ethanol concentration modify the physical properties of the solvent and influence the phenolic and aroma compounds extraction, colour, and acidity of the obtained samples. The higher content of phenolic compounds was determinate in the samples obtained in the darkness. The amounts of phenols were in the range of some red fruit liqueurs or walnut liqueurs.

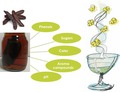

## 1 INTRODUCTION

Due to the chemical and nutritive composition of its fruit, the carob (*Ceratonia siliqua* L.) has multiple uses in the food and beverage production (Benković, Bosiljkov, Semić, Ježek, & Srečec, 2019). Also, the carob pod extracts are researched as a new cheap raw material for fermentation and biotechnological production of bioethanol, organic acids, and enzymes (Yatmaz & Turhan, 2018). In the Mediterranean countries, where carob trees grow, carob liqueur is typical alcoholic drink (minimum 15% v/v of ethanol and 100 g/L of sugar) made of carob pods. Carob liqueur is produced by maceration of partially crushed carob pods in the hydroalcoholic base, with the addition of sugar. Maceration is a solid–liquid extraction technique employed to obtain alcoholic extracts of varied type of vegetable matrices (citrus peels, flowers, leaves, medicinal/aromatic herbs). During the maceration process, various compounds that give a characteristic flavor and color as well as the biologically active compounds are extracted from the plant material into the hydroalcoholic base (Petrović, Vukosavljević, Đurović, Ntić, & Gorjanović, 2019; Rodríguez‐Solana, Salgado, Domínguez, & Cortés‐Diéguez, 2016; Rodríguez‐Solana, Vázquez‐Araújo, Salgado, Domínguez, & Cortés‐Diéguez, 2016; Veljović et al., 2019). Macerate, the solution obtained by the maceration process, could be used as a base for liqueurs or further distilled to give a distillate of macerate. This distillate contains volatile substances transferred from the plant material into the macerate (colored) and then into the distillate (colorless). The distillate can be added to the macerate for further flavor enrichment (Buglass & Caven‐Quantrill, 2012).

Despite the scarce scientific data on herbal/fruit liqueurs, there are some researches dealing with the development of these kinds of alcoholic drinks. Maceration parameters (type and strength of water‐alcohol base, maceration time, temperature, solid/liquid ratio) influence the quality of the macerate (Caldeira, Lopes, Delgado, Canas, & Anjos, 2018; Galego, Jockusch, & Da Silva, 2013; Gironés‐Vilaplana, Calín‐Sánchez, Moreno, Carbonell‐Barrachina, & García‐ Viguera, 2015; Jovanović et al., 2017; Rodríguez‐Solana, Coelho, et al., 2019; Rodríguez‐Solana, Salgado, Pérez‐Santín, & Romano, 2019; Rodríguez‐Solana, Vázquez‐Araújo, et al., 2016). For example, at higher alcohol percentage essential oils, lipids, and resins are dissolved, while at lower alcohol percentage substances soluble in water (organic acids, bitter substances, and carbohydrates) are dissolved. There are only a few scientific researches on carob‐based alcoholic drinks and on the nutritional characteristics of the carob liqueur (Rodríguez‐Solana, Carlier, Costa, & Romano, 2018; Rodríguez‐Solana, Salgado, et al., 2019). Rodríguez‐Solana, Salgado et al. (2019) investigated the process of maceration of carob in order to extract as many of the bioactive compounds as possible. The results showed that gallic acid is the most abundant bioactive compound in the carob liqueur, and the amount of both gallic acid and total phenolic compounds significantly dependent on the carob cultivar characteristics. In addition to phenolic compounds during maceration, other components of the carob pod are extracted into the macerate. Research by Rodríguez‐Solana et al. (2018) showed that carob liqueur is rich in minerals: Na, K, Ca, Mg, Cu, and Fe were detected in all liqueur samples. The most common ones were potassium, sodium, and calcium.

The available scientific literature does not define the optimal parameters of the maceration process for the production of carob liqueur. The aim of this study was to investigate the influence of the maceration process parameters on physicochemical characteristics of carob macerates and to determine the aroma compounds that are characteristic for this type of alcoholic drink. The maceration time (1–12 weeks), the ratio of the plant:hydroalcoholic base/solid:liquid (1:5 and 1:10), alcoholic strength (30%, 50% and 70%), and exposure to sunlight or darkness were the studied parameters. In addition, total phenolic content, antioxidant activity, total sugar content, pH value, and chromatic parameters of the carob macerates were determined.

## 2 MATERIALS AND METHODS

### 2.1 Reagents and standards

Folin–Ciocalteu (FC) reagent, iron (III) chloride hexahydrate, and potassium hexacyanoferrate (II) trihydrate were supplied by Kemika. Sodium carbonate, sodium chloride, sodium acetate, and zinc sulfate heptahydrate were of analytical grade, obtained from Gram‐mol. Acetic acid (glacial) was obtained from Carlo Erba Reagents (Barcelona, Spain) and hydrochloric acid (37%) from Fisher Scientific. Trolox (6‐hydroxy‐2,5,7,8‐tetramethylchromane‐2‐carboxylic acid), 2,4,6‐tripyridyl‐S‐triazine (TPTZ) as well as all analytical standards (gallic acid, sucrose, glucose, and fructose) were supplied by Sigma‐Aldrich. Ethanol 96% (v/v) was obtained from Kefo.

### 2.2 Preparation of carob macerates

Carob macerates were prepared by the maceration of chopped carob pods (4 cm size pieces, unroasted) in hydroalcoholic base (mix of 96% synthetic ethanol and water). Carob pods were macerated in 500 ml of hydroalcoholic base (30, 50, and 70% v/v of ethanol) in different solid to liquid ratio (1:5 and 1:10) at room temperature exposed to sunlight as well to darkness. During the maceration, the samples were daily manual shaken. Samples were obtained after every week during 12 weeks of maceration. Carob pods were obtained from local producer from the island of Vis, Dalmatia, Croatia.

### 2.3 HPLC analysis of extracted sugars

Concentrations of extracted sugars (sucrose, glucose, and fructose) in macerates at the end of maceration were determinate by HPLC. Prior the analysis, Carrez reagents were added to the sample and the precipitated proteins were removed by filtration (Lefebvre, Gabriel, Vayssier, & Fontagne‐Faucher, 2002). Obtained supernatants were filtered through 0.22‐mm nylon syringe filter (LAB Logistics Group GmbH, USA) and used for HPLC analysis on chromatograph (CLASS‐VP LC‐10A VP; Shimadzu, Kyoto, Japan) equipped with ion‐exchange column Supelcogel™ C‐610 H (L × i.d. = 30 cm × 7.8 mm) and guard column Supelcogel™ H (L × i.d.=5 cm × 4.6 mm), as well as refractive index detector. Separation and elution were performed using phosphoric acid (0.1% w/w) as the mobile phase at a flow rate of 0.5 ml/min (Trontel, Baršić, Slavica, Šantek, & Novak, 2010). The column and the refractive index detector were maintained at 30°C. The sample injection volume was 10 μl. Qualitative and quantitative determination was based on the injection of pure standards (sucrose, fructose, and glucose). Quantification was done by external calibration preparing calibration curves of six points with concentrations of standards (sucrose, fructose, and glucose) 0.1–5 g/L. Coefficient of determination (*R* ^2^) was .9989; .999, and .9993.

### 2.4 Determination of total phenolic content (TPC)

The total phenolic content (TPC) of each sample was determined by applying the Folin–Ciocalteu method (Singleton & Rossi Junior, 1965). Briefly, 0.3 ml of diluted samples or standard solutions (gallic acid) were added to 6 ml of distilled water and 0.5 ml of the Folin–Ciocalteu reagent, mixed thoroughly and allowed to stand for 5 min. Then, 1.5 ml of saturated sodium carbonate solution was added, mixed well, and filled to a total volume of 10 ml with distilled water. The samples were left at room temperature for 2 hr in the darkness. The absorbance of the samples was measured at 760 nm with an UV/Vis spectrophotometer (Heλios β, Unicam). The calibration curve used for the quantification of the samples was prepared with different concentrations of gallic acid standard solution (50–300 mg/L).

The total phenolic content was expressed as mg of gallic acid equivalents (GAE) per 100 ml of carob macerate.

### 2.5 Antioxidant activity

For the determination of antioxidative activity of carob macerates, ferric ion reducing antioxidant power (FRAP) was used. The FRAP assay was performed as previously described by Benzie and Strain (1999) with some modifications. FRAP reagent solution was made of the mixture of acetate buffering agent (300 mM, pH = 3.6), TPTZ (10 mM solution TPTZ in 40 mM HCl), and FeCl_3_ * 6H_2_O (20 mM) in volume ratio 10:1:1, respectively). The working FRAP reagent was prepared fresh on the day of the analysis. All samples, standards, and reagents were preincubated at 37°C. The examined sample (80 μl) was mixed with FRAP reagent (2080 μl) and distilled water (240 μl). After the reaction at 37°C for 5 min, the absorbance at 593 nm was measured. The standard curve was constructed by using serial dilution (0.1–2.0 mM) of Trolox stock solution. The final results were expressed as mM Trolox equivalents.

### 2.6 pH assay

The measurements of pH of all samples were done with Oakton pH 5 plus Meter pH meter (Oakton Instruments). The system was calibrated by placing pH‐probe in buffer pH 4.

### 2.7 Chromatic characteristics

The chromatic characteristics were determined on a Specord 50 Plus (Analytik Jena) and a 10‐mm glass cell, by measuring the transmittance of the sample every 10 nm from 380 to 770 nm, with a D65 illuminant. Based on the transmittance values, some parameters were calculated: luminosity (L*); saturation (C*); chromaticity coordinates (a* and b*), and hue (h*) (OIV – Compendium of International Methods of Analysis of Spirituous Beverages of Vitivinicultural Origin, 2014).

### 2.8 HS‐SPME and GC/MS conditions and analysis

Headspace (HS)‐solid‐phase microextraction (SPME) and gas chromatography (GC) were applied for analysis of aroma compounds. Aliquot of 8 ml of diluted sample (to the final concentration of 5% (v/v) ethanol) was pipetted into 20‐mL glass vial, spiked with 2 g NaCl, and capped with silicone septa. Manual sampling was done using 50/30 μm DVB/CAR/PDMS (divinylbenzene/carboxen/polydimethylsiloxane) 1 cm StableFlex fiber (Supelco), which is recommended for aroma compounds (volatiles and semivolatiles) (Câmara et al., 2007). Before use, fiber was conditioned according to manufacturer's instructions. After 10 min stabilization of the sample, fiber was exposed to the sample headspace for 40 min at 60°C with continuous magnetic stirring (Du, He, Li, Wanga, & Xiao, 2015). The SPME fiber was thermally desorbed in the programmed temperature vaporizer injector at 250°C during 5 min using splitless mode.

Gas chromatography instrument (GC 6890) equipped with MS detector (5973 Inert Mass Selective Detector) (Agilent Technologies Network, Santa Clara, CA, SAD) was used for aroma compounds analysis. Separations were performed using a 20 m × 0.18 mm (i.d.) capillary column coated with a 0.18 μm film of Rxi‐5 ms stationary phase (5%‐phenyl‐arylene‐95%‐dimethylpolysiloxane). Helium was used as a carrier gas with a 0.78 ml/min column flow rate and 3.8 ml/min total flow rate. The analyses of the SPME samples were performed using GC/MS under following conditions: The oven program temperature used was 5 min at 40°C, 40–160°C at a rate of 4°C/min, and 160–240°C at a rate of 10°C/min, with a final temperature hold for 3 min, resulting in total run of 46 min. The interface temperature for GC‐MS was 260°C. Temperature of ion source was 250°C. Mass spectra were acquired in the electron impact mode (70 eV) using full scan with a mass acquisition range of 30–450 m/z. Identification of aroma compounds was made on the basis of mass spectral libraries (NIST 47, NIST 147 and Wiley 175) as well as from literature data. All analyses were performed in duplicate.

### 2.9 Statistical analysis

Statistical analysis was carried out using the MS Excel tool XLStat (Addinsoft) and Statistica 12 (Statsoft Inc.) programs. A basic descriptive statistical analysis was followed by an analysis of variance (ANOVA) test for mean comparisons. Principal component analysis (PCA) was used to visualize the differences between carob macerates obtained after 12 weeks of carob pod maceration, based on physicochemical characteristics and aroma compounds.

## 3 RESULTS

### 3.1 Total phenolic content (TPC), antioxidant activity (FRAP), total sugar content, pH, and color parameters of carob macerates

Starting from the idea of using carob pods for preparing new added‐value liqueur, the present study was focused to determine and propose the optimal maceration process parameters for production of macerate with acceptable sensory and nutritional characteristics. The sample description as well as the total phenolic content (TPC), antioxidant activities, total sugar content, and pH value of carob macerates obtained after 12 weeks of maceration are shown in Table 1.

**Table 1:** Physicochemical characteristics of carob macerate obtained after 12 weeks of carob pod maceration in various strength of alcohol base (30, 50 and 70% v/v) and various solid/liquid ratio (1:5 = H and 1:10 = L) at room temperature exposed to sunlight (S) and darkness (D)

Alcohol base (% v/v)	Solid/Liquid ratio	Sunlight/Darkness	Sample name	Total phenols mg GAE/100 ml	FRAP mM Trolox‐a	Total sugars g/L	pH
30	1:5	S	30_SH	161.37 ± 5.61	18.47 ± 0.26	96.12 ± 3.56	5.52
		D	30_DH	201.27 ± 9.27	25.21 ± 0.05	107.09 ± 7.40	5.66
	1:10	S	30_SL	55.51 ± 7.07	5.36 ± 0.32	57.90 ± 0.58	5.67
		D	30_DL	91.24 ± 3.54	11.60 ± 0.42	59.50 ± 3.60	5.67
50	1:5	S	50_SH	147.71 ± 1.22	18.29 ± 0.91	98.17 ± 1.11	6.02
		D	50_DH	212.00 ± 3.90	25.32 ± 0.48	98.80 ± 0.57	5.99
	1:10	S	50_SL	59.66 ± 3.17	6.39 ± 0.35	56.84 ± 1.43	6.16
		D	50_DL	91.98 ± 1.10	11.43 ± 0.23	52.35 ± 4.30	6.04
70	1:5	S	70_SH	97.95 ± 0.24	13.24 ± 0.31	103.76 ± 3.70	6.16
		D	70_DH	155.39 ± 0.61	19.89 ± 0.66	101.80 ± 4.96	6.17
	1:10	S	70_SL	55.02 ± 4.88	6.53 ± 0.15	50.04 ± 2.90	6.27
		D	70_DL	76.37 ± 1.58	8.88 ± 0.11	50.36 ± 3.60	6.33

The results confirm the migration process of bioactive compounds, aroma compounds, and sugars flowing from the carob pod to the hydroalcoholic base. Extracted compounds are responsible for the characteristic flavor (taste and aroma), color, and functional properties of carob macerates. Based on obtained results, there are differences in physicochemical characteristics of the macerates. The results are subjected to multivariate statistical analysis to evaluate the significance of the tested parameters.

**Figure 1:**
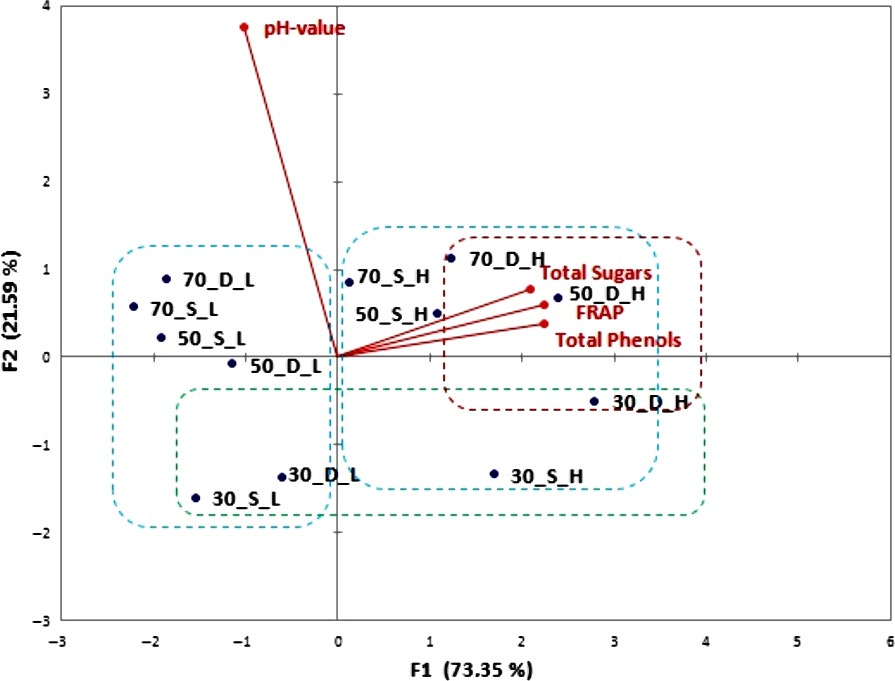
Principal component analysis (PCA) score plot for carob macerates obtained after 12 weeks of carob pod maceration in various strength of alcohol base (30, 50, and 70% v/v) and various solid/liquid ratio (1:5 = H and 1:10 = L) at room temperature exposed to sunlight (S) and darkness (D), based on physicochemical characteristics

**Figure 2:**
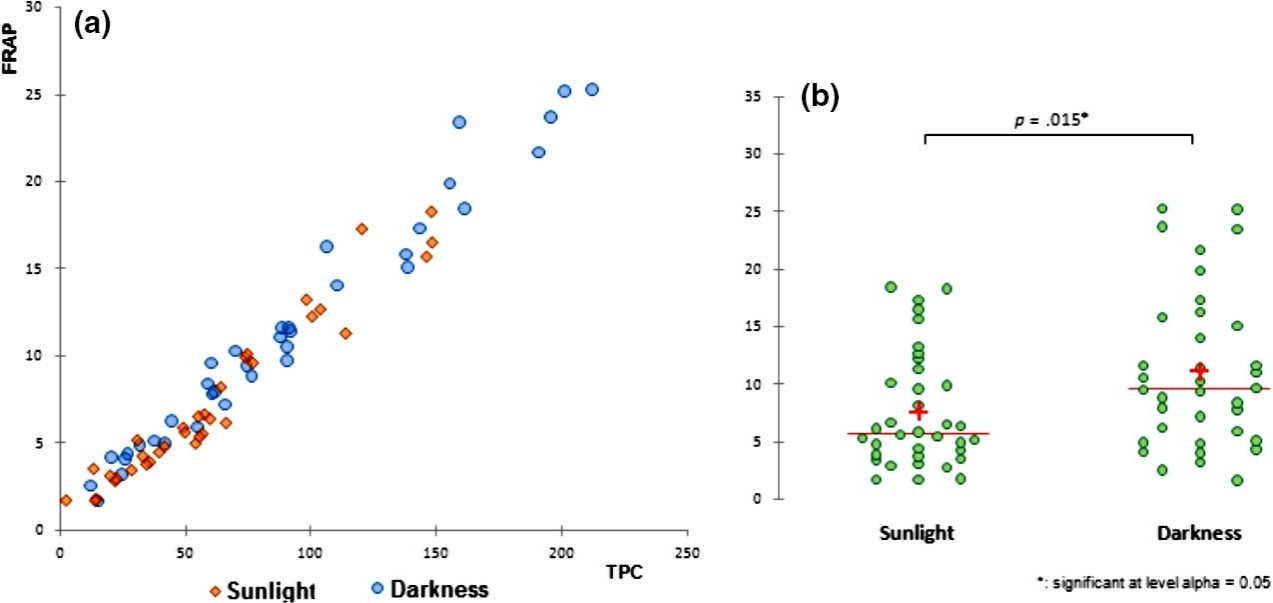
Positive correlation between the carob macerates TPC and the antioxidant activity determined by FRAP assay (a) and the difference between the antioxidant activity of macerates produced in sunlight and darkness (b)

**Figure 3:**
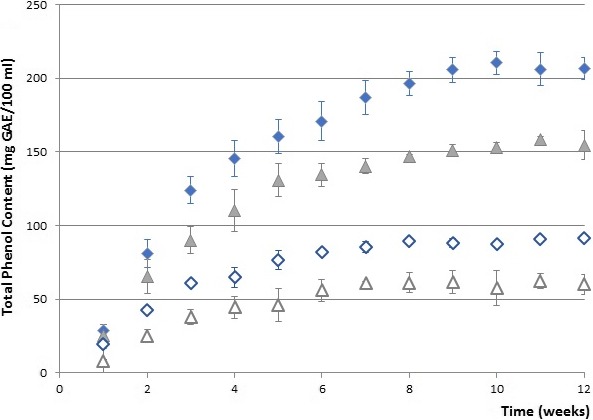
Total phenols solid–liquid extraction (ratio 1:5—filled symbols and 1:10—open symbols) during carob pod maceration in 50% v/v hydroalcoholic base at room temperature exposed to sunlight (triangle) as well to darkness (rhomb)

Figure 1 shows the biplot from the first two principal components which accounted for almost 95% of the total variance between the samples (PC1 [73.35%] and PC2 [21.59%]). The samples were divided into three main groups according to following parameters. Extracted carob components (sugars and polyphenols in an approximately equal relationship) contribute to the first component PC1, and the samples are distributed on the left and right sides of the horizontal axis and divided into two groups depending on the solid/liquid ratio. Within the right group, subgroup of samples macerated in the dark can be detected (marked red). Value with the most influence on PC2 component was pH value (92.7%) and macerates prepared with 30% alcoholic strength (both with higher and lower carob quantity). These samples have the lowest pH value and make a third subgroup of separated samples (marked green). Sample 50_DH was closely correlated by TPC and sugar content, as well as FRAP value which indicates that these maceration conditions can be recommended for obtaining macerate of desired functional properties. Furthermore, there was a positive correlation (*R* = .915) between the carob macerates TPC and antioxidant activity determined by FRAP assay (Figure 2a). Statistically significant differences in TPC and consequently antioxidant activity were found between samples macerated in light and darkness (Figure 2b). The TPC expressed as mg of gallic acid equivalents (GAE)/100 ml for the macerates displayed a sharp increase in the first 6 weeks, keeping then a stable increase until the end of the study. The higher amount of phenolic compounds was found in the macerates obtained in the darkness. TPC ranging from about 20 mg GAE/100 ml after the first week of maceration up to about 200 mg GAE/100 ml after 12 weeks of maceration, depending also on the tested parameters (Figure 3).

**Figure 4:**
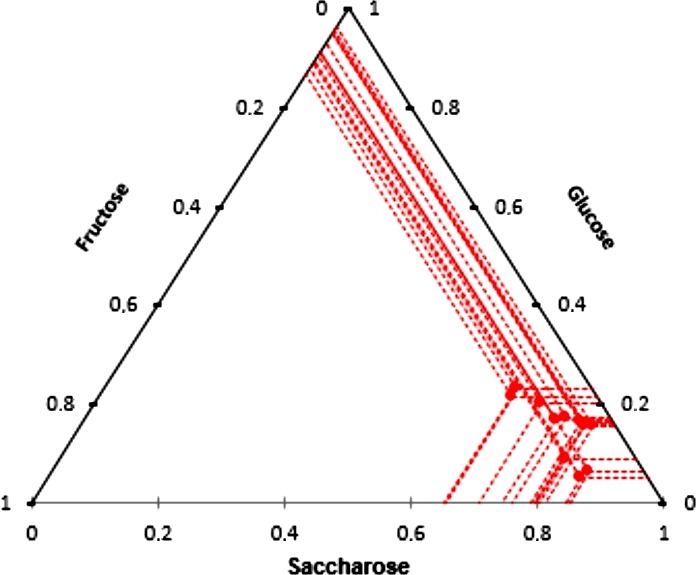
*Ternary diagrams* present the proportion of individual sugars (glucose, fructose, and saccharose) in macerates obtained after a 12 week of carob pod maceration

In addition to phenolic compounds, during maceration, other carob compounds are extracted into the macerate. Sugar analysis showed that macerates containing, in average, values ranging between 96 and 107 g/L of total sugars in the case of higher carob quantity, while for the lower quantity the concentration is expectedly, half less. In the total sugar content, the most abundant were sucrose (≈75%), glucose (≈15%), and fructose (≈10%), (Figure 4). The exposure to sunlight did not have a significant impact on the amount of extracted sugars as well as the strength of the alcohol for the higher quantity of carob (Figure 1). In macerates with the lower carob quantity, the lowest sugar concentration was measured at high alcohol strength, which can be explained by the higher solubility of the polar sugar molecules in water. At higher carob quantity, a higher amount of sugar could be extracted and equilibrium was achieved independently of the alcohol strength. According to the legislation, a liqueur should have at least 100 g/L of sugar, approximately obtained by carob maceration in hydroalcoholic base in the ratio 1:5.

**Figure 5:**
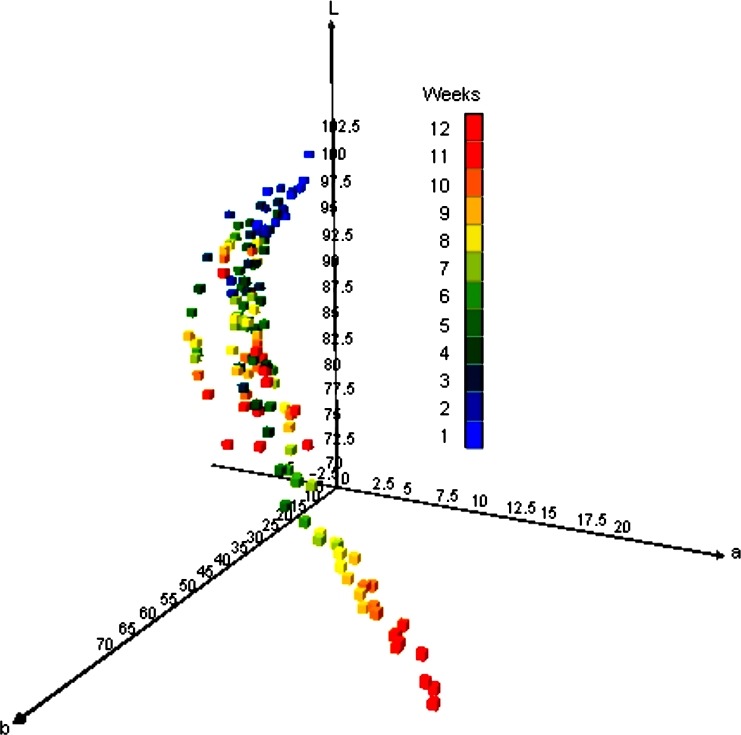
Changes of the chromate characteristics of macerates during 12 weeks of carob pod maceration

**Figure 6:**
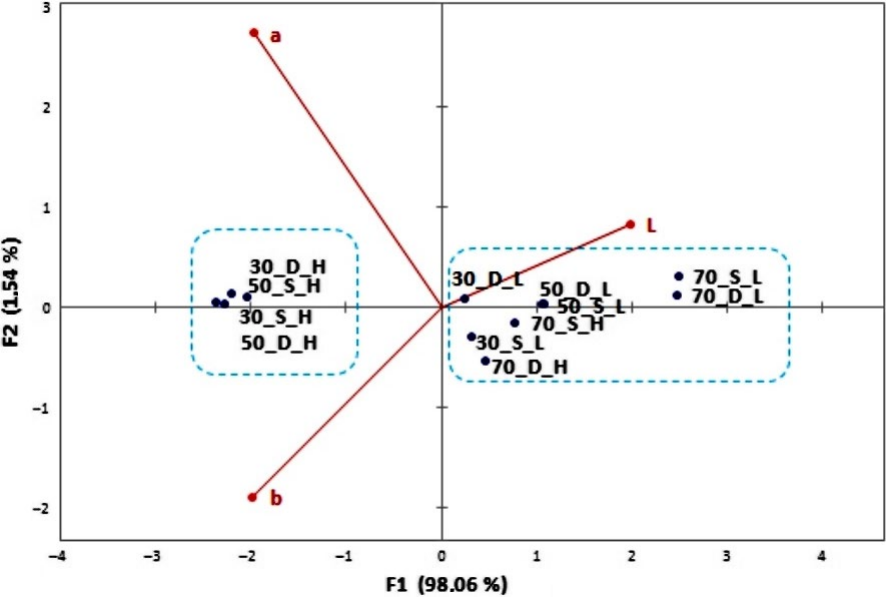
Principal component analysis (PCA) score plot for carob macerates obtained after 12 weeks of carob pod maceration in various strength of alcohol base (30, 50, and 70% v/v) and various solid/liquid ratio (1:5 = H and 1:10 = L) at room temperature exposed to sunlight (S) and darkness (D), based on chromatic characteristics

The results of macerates color parameters during the 12 weeks period are presented in Figure 5. There was a decrease in all macerates luminosity (*L*‐values) which ranged from 96.6 ± 1.8 at the beginning of maceration process to an 80.9 ± 7.5 at the end of the process. The *b* values increasing during the time which implied color fluctuation to more yellow, while the *a* values change from the negative to the positive value implied the change of color from green to red. According to the final chromatic values, obtained carob macerates expressed the color close to the orange/brown range. In terms of color, the biplot from the first two principal components is presented on Figure 6. Two differentiated groups were observed in Figure 6: One of them can be distinguished by the highest proportion of carob pods (1:5 = H) macerated in 30% and 50% v/v of alcohol.

### 3.2 Aroma compounds characterization of carob macerates

A total of 95 aroma compounds have been identified in carob macerates. Twenty‐seven components were detected in all samples, of which 17 esters, 3 alcohols, 4 ketones, and 2 acids (Table 2). These compounds constituted 70%–85% of the total carob macerates flavor. Low molecular weight ethyl esters, ethyl hexanoate, ethyl 2‐methyl propanoate, ethyl octanoate, ethyl benzoate, ethyl butanoate, and ethyl cinnamate, were the most abundant, followed by high molecular weight esters ethyl hexadecanoate, propan‐2‐yl hexadecanoate, ethyl (Z)‐octadec‐9‐enoate and propan‐2‐yl tetradecanoate. In addition, ethyl pentanoate was detected in all samples, with the exception of sample 30_SL and n‐amyl acetate in all samples, with the exception of samples 70_DL and 50_DL. Free acids detected in all samples were decanoic and dodecanoic acids, especially at higher concentrations in samples with 30% v/v ethanol as solvent. Additionally, tetradecanoic and hexadecanoic acid as free acids were detected in traces in some samples. Except acids and esters, some alcohols and ketones were detected in all samples. Nonan‐1‐ol, nonan‐2‐ol, and undecan‐2‐ol fatty alcohols are widespread in nature and occur in many fruits, spices, and essential oils with pleasant aroma as well as ketones undecan‐2‐one and pentadecan‐2‐one (National Center for Biotechnology Information).

**Table 2:** Average peak areas (%) of volatile aroma compounds that occur in all samples of carob macerate obtained after 12 weeks of carob pod maceration in various strength of alcohol base (30, 50 and 70% v/v) and various solid/liquid ratio (1:5‐High and 1:10‐Low) at room temperature exposed to sunlight and darkness

Compounds	Rt (min)	Q‐match quality	70_H	70_L	50_H	50_L	30_H	30_L	70_H	70_L	50_H	50_L	30_H	30_L
			Sunlight						Darkness					
Esters														
Ethyl 2‐methylpropanoate (*Ethyl isobutyrate*)	3.04	95	12.56 ± 1.46	6.66 ± 0.03	6.37 ± 0.10	10.06 ± 0.36	5.02 ± 0.07	6.96 ± 0.80	11.16 ± 0.01	7.49 ± 0.32	6.36 ± 0.34	9.20 ± 0.62	6.11 ± 0.41	6.28 ± 0.17
Ethyl butanoate (*Ethyl butyrate*)	4.18	94	2.55 ± 0.27	2.52 ± 0.24	2.53 ± 0.12	2.25 ± 0.38	1.86 ± 0.18	2.44 ± 0.22	2.45 ± 0.21	2.43 ± 0.001	2.42 ± 0.05	1.97 ± 0.40	1.96 ± 0.07	2.10 ± 0.13
Ethyl 2‐methylbutanoate (*Ethyl2‐methylbutyrate*)	5.64	95	0.99 ± 0.12	0.41 ± 0.006	0.17 ± 0.09	0.85 ± 0.02	0.15 ± 0.05	0.24 ± 0.06	1.02 ± 0.05	0.50 ± 0.05	0.20 ± 0.00	0.69 ± 0.11	0.28 ± 0.04	0.18 ± 0.09
Ethyl hexanoate	12.41	97	28.30 ± 1.08	36.94 ± 1.93	39.97 ± 0.93	29.23 ± 1.21	34.16 ± 1.24	37.26 ± 1.43	28.73 ± 1.12	38.46 ± 0.44	40.03 ± 1.54	28.79 ± 0.76	34.73 ± 1.42	38.61 ± 1.63
Ethyl heptanoate (*Cognac ester*)	16.05	98	0.69 ± 0.001	1.12 ± 0.05	0.79 ± 0.05	0.72 ± 0.001	0.61 ± 0.04	0.95 ± 0.12	0.77 ± 0.06	1.15 ± 0.02	0.72 ± 0.11	0.70 ± 0.03	0.90 ± 0.04	0.71 ± 0.05
Ethyl octanoate	19.77	97	4.75 ± 0.41	11.53 ± 0.36	7.74 ± 0.61	5.92 ± 0.55	8.29 ± 0.14	12.96 ± 0.16	5.96 ± 0.05	10.56 ± 0.006	7.76 ± 0.46	6.44 ± 0.28	11.86 ± 0.26	9.07 ± 0.55
Ethyl (E)‐oct‐3‐enoate	20.05	97	0.34 ± 0.03	1.38 ± 0.47	1.28 ± 0.41	0.39 ± 0.02	0.69 ± 0.07	0.81 ± 0.09	0.37 ± 0.01	1.29 ± 0.03	1.69 ± 0.13	0.37 ± 0.01	0.78 ± 0.01	0.81 ± 0.05
Ethyl benzoate	20.76	91	5.71 ± 1.25	3.87 ± 0.37	3.99 ± 0.47	5.09 ± 1.27	3.08 ± 0.14	3.55 ± 0.46	6.68 ± 0.90	4.36 ± 0.25	5.22 ± 0.14	6.05 ± 0.28	3.95 ± 0.35	4.37 ± 0.261
Ethyl 3‐hydroxydodecanoate	25.27	81	0.91 ± 0.37	0.35 ± 0.02	0.27 ± 0.05	0.22 ± 0.10	0.14 ± 0.002	0.18 ± 0.02	1.12 ± 0.17	0.43 ± 0.08	0.35 ± 0.07	0.87 ± 0.19	0.35 ± 0.06	0.24 ± 0.02
Ethyl (E)‐dec‐4‐enoate	26.12	99	0.28 ± 0.13	0.95 ± 0.01	0.36 ± 0.01	0.12 ± 0.12	0.52 ± 0.07	0.45 ± 0.02	0.12 ± 0.05	0.61 ± 0.003	0.39 ± 0.05	0.24 ± 0.06	0.48 ± 0.09	0.33 ± 0.003
Ethyl decanoate	26.30	97	0.52 ± 0.09	1.28 ± 0.02	1.40 ± 0.48	0.61 ± 0.32	0.36 ± 0.09	1.04 ± 0.23	0.47 ± 0.08	1.05 ± 0.07	0.67 ± 0.04	0.45 ± 0.04	1.79 ± 0.15	0.69 ± 0.05
(Z)‐4‐dodecoxy‐4‐oxobut‐2‐enoic acid	28.83	94	3.10 ± 1.14	1.42 ± 0.60	0.88 ± 0.02	1.63 ± 0.72	0.83 ± 0.27	1.00 ± 0.06	1.33 ± 0.44	0.52 ± 0.08	0.19 ± 0.05	1.89 ± 0.06	1.75 ± 0.19	1.16 ± 0.89
Ethyl (E)‐3‐phenylprop‐2‐enoate (*Ethyl cinnamate*)	31.37	95	2.12 ± 0.88	1.92 ± 0.35	1.39 ± 0.57	2.35 ± 0.89	2.09 ± 0.44	2.03 ± 0.86	2.79 ± 0.53	2.24 ± 0.29	2.11 ± 0.56	3.29 ± 0.68	2.39 ± 0.23	2.08 ± 0.10
Propan‐2‐yl tetradecanoate (*Isopropyl Myristate*)	37.45	99	0.39 ± 0.22	0.41 ± 0.24	0.54 ± 0.20	0.51 ± 0.31	0.47 ± 0.09	0.18 ± 0.04	0.26 ± 0.26	0.14 ± 0.14	0.21 ± 0.01	0.24 ± 0.16	0.17 ± 0.17	0.28 ± 0.03
Ethyl hexadecanoate (*Ethyl palmitate*)	39.99	95	1.25 ± 0.33	0.85 ± 0.42	2.27 ± 0.06	0.95 ± 0.09	2.04 ± 0.06	0.09 ± 0.03	0.96 ± 0.36	1.69 ± 0.68	2.05 ± 0.92	0.42 ± 0.35	1.46 ± 0.43	2.09 ± 0.80
Propan‐2‐yl hexadecanoate (*Isopropyl Palmitate*)	40.19	83	1.04 ± 0.21	2.25 ± 1.02	1.87 ± 0.65	1.72 ± 0.17	4.13 ± 0.96	0.93 ± 0.14	3.25 ± 1.38	0.85 ± 0.44	1.64 ± 0.09	1.33 ± 0.47	1.14 ± 0.19	1.37 ± 0.41
Ethyl (Z)‐octadec‐9‐enoate (*Ethyl Oleate*)	42.02	99	0.24 ± 0.09	0.56 ± 0.19	0.67 ± 0.26	0.25 ± 0.01	0.60 ± 0.05	0.31 ± 0.02	0.37 ± 0.02	0.73 ± 0.18	0.89 ± 0.60	0.33 ± 0.03	0.61 ± 0.31	0.95 ± 0.53
Alcohols														
Nonan‐2‐ol	15.85	86	1.29 ± 0.15	0.75 ± 0.15	0.49 ± 0.02	1.45 ± 0.08	0.41 ± 0.04	0.61 ± 0.24	1.66 ± 0.002	0.89 ± 0.03	0.56 ± 0.01	1.24 ± 0.03	0.72 ± 0.03	0.49 ± 0.15
Nonan‐1‐ol	18.80	90	0.40 ± 0.01	0.30 ± 0.05	0.31 ± 0.01	0.43 ± 0.01	0.13 ± 0.02	0.23 ± 0.02	0.47 ± 0.12	0.39 ± 0.01	0.25 ± 0.004	0.39 ± 0.01	0.19 ± 0.02	0.19 ± 0.05
Undecan‐2‐ol	22.99	87	1.24 ± 0.29	1.19 ± 0.12	1.01 ± 0.08	1.27 ± 0.34	0.85 ± 0.19	1.08 ± 0.06	1.81 ± 0.30	0.81 ± 0.11	0.99 ± 0.03	1.39 ± 0.24	1.34 ± 0.004	0.41 ± 0.03
Ketones														
Nonan−2‐one	16.31	97	1.06 ± 0.13	1.19 ± 0.13	1.28 ± 0.14	1.28 ± 0.01	0.99 ± 0.20	1.04 ± 0.13	1.22 ± 0.14	1.19 ± 0.08	1.62 ± 0.13	1.06 ± 0.02	1.09 ± 0.001	1.11 ± 0.21
(E)‐non‐3‐en‐2‐one	18.71	92	0.09 ± 0.02	0.23 ± 0.02	0.26 ± 0.003	0.10 ± 0.004	0.18 ± 0.003	0.12 ± 0.005	0.23 ± 0.04	0.17 ± 0.004	0.33 ± 0.04	0.21 ± 0.06	0.26 ± 0.002	0.28 ± 0.04
Undecan‐2‐one	23.45	94	0.28 ± 0.08	1.25 ± 0.02	1.47 ± 0.13	0.40 ± 0.12	1.07 ± 0.15	0.95 ± 0.03	0.42 ± 0.01	1.49 ± 0.08	1.76 ± 0.01	0.53 ± 0.005	1.24 ± 0.004	1.45 ± 0.07
Pentadecan‐2‐one	35.44	91	0.08 ± 0.09	0.76 ± 0.06	1.10 ± 0.14	0.36 ± 0.05	0.87 ± 0.24	0.65 ± 0.13	0.15 ± 0.02	0.94 ± 0.02	1.08 ± 0.19	0.18 ± 0.02	0.69 ± 0.02	0.90 ± 0.23
Acids														
Decanoic acid	25.97	96	0.78 ± 0.30	0.68 ± 0.50	0.11 ± 0.101	0.35 ± 0.10	0.83 ± 0.38	0.35 ± 0.10	0.21 ± 0.10	0.76 ± 0.11	0.35 ± 0.04	0.25 ± 0.03	0.20 ± 0.01	0.88 ± 0.38
Dodecanoic acid (*Lauric acid*)	31.72	96	0.69 ± 0.36	0.30 ± 0.08	0.12 ± 0.06	0.64 ± 0.35	0.46 ± 0.03	0.44 ± 0.15	0.42 ± 0.07	0.30 ± 0.06	0.25 ± 0.01	0.52 ± 0.11	0.34 ± 0.01	0.25 ± 0.01

Note

Data are presented as the means of duplicate measurements ± *SD*.

**Figure 7:**
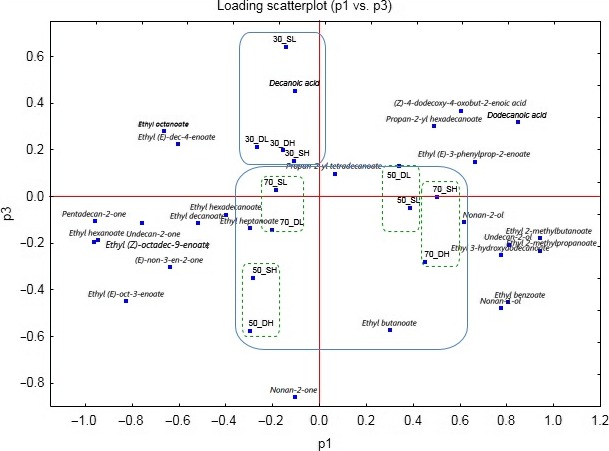
A biplot representation of the aroma compounds of carob macerates according to the principal component analysis

Multivariate analysis was used to visualize the relation between aroma compounds in the complete dataset of different carob macerates. The separation was based on the applied multivariate tool principal component analysis (PCA), which resulted in a reduced dimension plot, showing separation of aroma compounds in different samples. In the shown biplot (Figure 7), one can see the grouping of samples of the same alcoholic strength regardless of whether the samples are macerated in sunlight or darkness. On the other hand, the alcohol base strength affects the presence of certain aromas in macerates. This can be best seen on macerates of higher strength alcohol as a base with a high carob quantity that are rich in water‐insoluble compounds such as ethyl benzoate, ethyl 2‐methylbutanoate, or undecan‐2‐one. It can be noticed that higher alcoholic strength macerates are more abundant with the aromatic compounds than the lower alcoholic strengths macerates. Given the results and results of the physicochemical analysis, the 50% v/v alcoholic strength base can be considered optimal for obtaining macerate of desired characteristics.

## 4 DISCUSSION

Recently, researchers have focused on the valorization of carob pods since they are an excellent source of sugar (Boublenza et al., 2017; Bulca, 2016; Mazaheri et al., 2012; Turhan, Bialka, Demirci, & Karhan, 2010) as well as bioactive compounds such as polyphenols which can be efficiently extracted by the maceration of chopped carob pods in hydroalcoholic base (Goulas, Stylos, Chatziathanasiadou, Mavromoustakos, & Tzakos, 1875; Nasar‐Abbas et al., 2016; Rodríguez‐Solana, Salgado, et al., 2019). Obtained liqueur is a desirable, aromatic drink with potential health benefits, and the amount of extracted compounds depends on the maceration conditions (Rodríguez‐Solana, Coelho, et al., 2019; Rodríguez‐Solana, Salgado, et al., 2019). The highest TPC values were found in the samples in which carob was macerated in lower strength alcoholic base (50% v/v and 30% v/v) and exposed to darkness. Amounts of phenolics were in the range of some red fruit liqueurs and walnut liqueurs (Mrvčić et al., 2012; Sokół‐Łętowska et al., 2014). Regarding alcoholic strength, results are in accordance with Cavdarova and Makris (2014) who tested a series of acidified and nonacidified hydroalcoholic solvent systems for their efficiency to recover polyphenolics from carob powder. Solutions with 30% of ethanol acidified with either acetic or citric acid, and displayed the highest phenolic concentration, more than in samples with higher alcoholic strength. Jakopic et al. (2007) concluded that content of total phenolics in walnut liqueur increased with the ethanol concentration. The lowest values were detected at 40% of ethanol and the highest at 60%, but this does not hold for analyzed individual phenolic compounds. Like in our carob macerate, gallic acid was one of the major analyzed phenolic substances in the walnut liqueur, and its concentration was highest at 40% ethanol and decreased when the proportion of ethanol was increased. The same trend also shows the catechin and syringic acid. In contrast, Rodríguez‐Solana, Vázquez‐Araújo, et al. (2016) showed that the highest extraction of total phenols from aromatic and medicinal herbs is obtained with high ethanol concentration (70%). Changes in ethanol concentration modify the physical properties of the solvent and may influence the extraction of phenolic compounds. According to literature, carob pods contain 448 mg/kg extractable polyphenols comprising gallic acid, hydrolyzable and condensed tannins, flavonol glycosides, and traces of isoflavonoids (Goulas et al., 1875). Carob fruit is one of the richest sources of gallic acid. Tannins comprise the most characteristic group of polyphenols in carob fruits and contribute to their astringency. The carob flour production process has an important influence on the polyphenolic patterns, and roasted carob products contain the highest levels of gallic acid. Rodríguez‐Solana, Salgado, et al. (2019) showed noticeable phenolic and antioxidant differences between liqueur prepared with different carob varieties and depending of flour particle size. Furthermore, concerning the macerates, there was a strong positive correlation between the TPC and the antioxidant activity determined by FRAP assay (Figures 1 and 2) which indicate the significant contribution of the carob phenolic compounds to macerates antioxidant activity. Link between phenolic compounds and antioxidant activity in spirit drinks, herbal distillates, and liqueurs were also proved by other authors (Andreou et al., 2018; Mrvčić et al., 2012). These results also indicate a higher amount of phenolic compounds in the macerates obtained in the darkness. Phenolic compounds are described in the literature as very unstable and highly susceptible compounds to degradation where numerous factors like light, temperature, oxygen, or pH value influence their stability. Reduction in the TPC is especially pronounced at higher temperatures and at the presence of light (Del‐Toro‐Sánchez et al., 2015). These are exactly the conditions in which liqueurs are traditionally produced. Namely, many of the liqueurs are home‐made fruit liqueurs produced with available agricultural raw materials, whose production are in accordance with some traditional recipes. These recipes commonly suggest fruit maceration in the sunlight, most likely due to faster extraction at higher temperatures (Paz, Fernández, Matías, & Pinto, 2014). In alignment to our research, it should be highlighted that such conditions should be avoided and these habits need to be changed due the sensitivity and degradation of phenolic compounds exposed to sunlight and in order to preserve liqueur antioxidant potential. Also, good manufacturing practice should be overwritten from wineries. Wine is filled in colored bottles which provide some protection from UV and visible light radiation, while liqueurs are usually filled in clear glass bottles, often in attractive shapes.

The strength of alcohol proved to be the most important parameter in phenolic and aroma compounds extraction, color, and acidity of macerates. The macerates prepared in 30% hydroalcoholic base had higher total acidity than the macerates prepared with higher alcoholic strength (50% and 70%). Acidity is also affected by the carob pod quantity. The macerates prepared with higher quantity of carob had higher total acidity than those prepared with lower quantity of carob (Figure 1). The increase of acidity in macerate that has a higher amount of carob as well as in macerate with lower alcoholic strength can be explained by the presence of acid in carob pods and better solubility of acid substances in water than in ethanol. GC/MS analysis of macerates revealed that the main free acids were decanoic and dodecanoic acid, detected in all samples. Furthermore, other free acids like tetradecanoic and hexadecanoic acid are detected in traces in some samples. Detected acids among others contribute not only to the total acidity of the samples but also to the overall flavor and aroma. Namely, Farag and El‐Kersh (2017) detected short‐chain fatty acids like pentanoic acid (15%–25%) and hexanoic acid (20%) as the main components in carob pod volatiles. Several other less abundant acids were detected including pyruvic, isobutyric, butyric, heptanoic acid, octanoic, and benzoic acids.

The strength of alcohol and carob pod quantity proved to be important parameters in a final macerates color (Figure 6). The macerates prepared with higher quantity of carob as well as with lower alcoholic strength had lower luminosity due to the particles (phenolics, minerals, acids, sugars) that can pass from the carob to macerate during the maceration, increasing the turbidity (Cavdarova & Makris, 2014; Gironés‐Vilaplana et al., 2015). Namely, the prepared macerates ultimately constitute very complex matrixes with a range of natural compounds which have been previously identified in the phytochemical profile of carob (Nasar‐Abbas et al., 2016) and which are soluble in the hydro‐alcohol base used. Different from the TPC and antioxidant activity, the influence of sunlight on the color parameters was not recorded.

It should be noted that although there are some common guidelines, optimal parameters for the production of liqueurs from different fruits may be different. Nour, Trandafir, and Central (2015) optimized the hydroalcoholic extraction conditions to maximize the anthocyanin content, total phenolic content, and antioxidant activity of bilberry extracts in order to obtain bilberry liqueur. Ethanol concentration had the main impact on the extraction efficiency, and the optimum extraction conditions are ethanol concentration 91.83%, solid to liquid ratio 1.22, and extraction time 23.5 days. Furthermore, similar process parameters for maceration of different parts of selected aromatic and medicinal plants (flowers, seeds, roots, and leaves) in grape marc distillates were investigated by Rodríguez‐Solana, Vázquez‐Araújo, et al. (2016). Based on TPC, color parameters, and consumer preference, they selected the optimal maceration conditions: 70% v/v of ethanol, 40 g/L plant concentration, and 3 weeks of maceration process, significantly different from our optimum conditions for the carob. Therefore, these results indicate the importance of another maceration parameters: the plant/fruit particle size and its structure because they have important influence on compounds extraction efficiency and consequently duration of the maceration. Using dry, leathery, and larger sized chopped carob pods (4 cm) in our research, longer extraction time is needed to obtain macerate with the highest TPC.

Detection of aroma compounds is one of the most important steps in the evaluation of spirits, liqueurs, and other types of alcoholic beverages quality (Chen, Capone, & Jeffery, 2019). The aroma is influenced by many factors, including the quality of the starting raw material together with variables within the production process (Sliwinska, Wisniewska, Dymerski, Wardencki, & Namiesnik, 2015). The volatile aromatic compounds are mostly esters, higher alcohols, and aldehydes. Identification of these compounds is important to establish the flavor characteristics of a given spirit drink (Hanousek Čiča et al., 2019). This is the first scientific report of the volatile aroma compounds in carob macerate. The volatile organic compounds of carob fruit and flour were previously reported by Krokou, Stylianou, and Agapiou (2019). The acids were the most dominant volatile organic class in both carob fruit and flour, followed by esters. The acids and esters were responsible for the 96% of the emitted volatile organic compounds in their experiments. Acids include the acetic acid, 2‐methylpropanoic acid, butanoic acid, and hexanoic acid, whereas from the class of esters are the methyl 2‐methylpropanoate, methyl butanoate, methyl hexanoate, and 2‐methylbutyl 2‐methylpropanoate. Among them, 2‐methylpropanoic acid was the most abundant. In our experiment, detectable acids are not considered as the main volatile components, but the ethyl esters of these acids. Ethyl esters were formed during extraction of acids from carob pods to the hydroalcoholic base. Ethyl 2‐methylpropanoate was present in approx. 10% of the total flavor. Nonan‐2‐one and heptanone were detected in carob fruit by Krokou et al. (2019). In our experiment, heptanone was detected in only 4 of 12 macerates (in macerates with higher alcohol strength and higher quantity of macerated carob). Nonan‐2‐one is a plant metabolite, present in many fruits and spices (National Center for Biotechnology Information). It is a clear slightly yellow liquid. It is most represented in the sample 50_DH and significantly contributes to the yellow color of this macerate (Figure 6). The esters generally have a pleasant aroma and a very intense odor, and they are important beverage aroma components (Lukić et al., 2011; Sliwinska et al., 2015). These compounds make a positive contribution to the general quality of the spirit, being responsible for their “fruity” and “floral” sensory properties (Câmara et al., 2007). Ethyl hexanoate presents a tropical fruit aroma and ethyl octanoate is associated with banana, pineapple, and brandy‐like aromas (Genovese, Ugliano, Pessina, Gambuti, & Piombino, 2004; Rogerson & De Freitas, 2002). As many volatile esters, ethyl benzoate has a pleasant odor described as sweet, wintergreen, fruity, medicinal, cherry, and grape. Ethyl butanoate, present in many fruits, has a fruity odor, similar to pineapple and ethyl 2‐methylpropanoate fruity, aromatic odor. Krokou et al. (2019) detected only 45 aroma compounds in carob fruit, significantly less than in our experiment, probably due to the application of SPME directly, without any solvent.

## 5 CONCLUSIONS

In this study, optimal maceration process parameters for production of the aromatic and bioactive rich carob macerate are determined. During the maceration process, macerates changed in aroma properties, color, sugar content, phenols composition, and antioxidant activity depending on studied parameters. Changes in ethanol concentration modify the physical properties of the solvent and affect the macerates composition. Carob pod maceration in 50% v/v hydroalcoholic base in darkness, in solid to liquid ratio 1:5 at room temperature, can be recommended as maceration conditions for obtaining macerate of desired functional properties, sweetness, and desirable aroma compounds. Optimal maceration time is 6–8 weeks. The total phenolic content was in the range of some red fruit liqueurs or walnut liqueurs, and sugars (mostly sucrose) ranging between 96 and 107 g/L. Ethyl esters, ethyl hexanoate, ethyl 2‐methylpropanoate, ethyl octanoate, ethyl benzoate, ethyl butanoate, and ethyl cinnamate, are the compounds found in greater proportion in the carob macerates flavor.

## CONFLICT OF INTEREST

The authors declare no conflict of interest.

## ETHICAL APPROVAL

This study does not involve any human or animal testing.
